# Clinician-Driven Design of *VitalPAD*–An Intelligent Monitoring and Communication Device to Improve Patient Safety in the Intensive Care Unit

**DOI:** 10.1109/JTEHM.2018.2812162

**Published:** 2018-03-05

**Authors:** Luisa Flohr, Shaylene Beaudry, K Taneille Johnson, Nicholas West, Catherine M Burns, J Mark Ansermino, Guy A Dumont, David Wensley, Peter Skippen, Matthias Görges

**Affiliations:** Faculty of MedicineThe University of British ColumbaVancouverBCV6T 1Z3Canada; Department of AnesthesiologyPharmacology and TherapeuticsThe University of British ColumbaVancouverBCV6T 1Z3Canada; Department of Systems Design EngineeringUniversity of WaterlooWaterlooONN2L 3G1Canada; BC Children’s Hospital Research InstituteVancouverBCV5Z 4H4Canada; Department of Electrical and Computer EngineeringThe University of British ColumbaVancouverBCV6T 1Z4Canada; Department of PediatricsThe University of British ColumbaVancouverBCV6H 3V4Canada

**Keywords:** Communication systems, decision support systems, human factors, patient monitoring, user centered design

## Abstract

The pediatric intensive care unit (ICU) is a complex environment, in which a multidisciplinary team of clinicians (registered nurses, respiratory therapists, and physicians) continually observe and evaluate patient information. Data are provided by multiple, and often physically separated sources, cognitive workload is high, and team communication can be challenging. Our aim is to combine information from multiple monitoring and therapeutic devices in a mobile application, the *VitalPAD*, to improve the efficiency of clinical decision-making, communication, and thereby patient safety. We observed individual ICU clinicians, multidisciplinary rounds, and handover procedures for 54 h to identify data needs, workflow, and existing cognitive aid use and limitations. A prototype was developed using an iterative participatory design approach; usability testing, including general and task-specific feedback, was obtained from 15 clinicians. Features included map overviews of the ICU showing clinician assignment, patient status, and respiratory support; patient vital signs; a photo-documentation option for arterial blood gas results; and team communication and reminder functions. Clinicians reported the prototype to be an intuitive display of vital parameters and relevant alerts and reminders, as well as a user-friendly communication tool. Future work includes implementation of a prototype, which will be evaluated under simulation and real-world conditions, with the aim of providing ICU staff with a monitoring device that will improve their daily work, communication, and decision-making capacity. Mobile monitoring of vital signs and therapy parameters might help improve patient safety in wards with single-patient rooms and likely has applications in many acute and critical care settings.

## Graphical abstract

The VitalPAD application will extract data from multiple bedside devices in order to provide an integrated patient monitoring tool. User-centered design helped refine the application prototype to optimize information layout for different clinical roles.

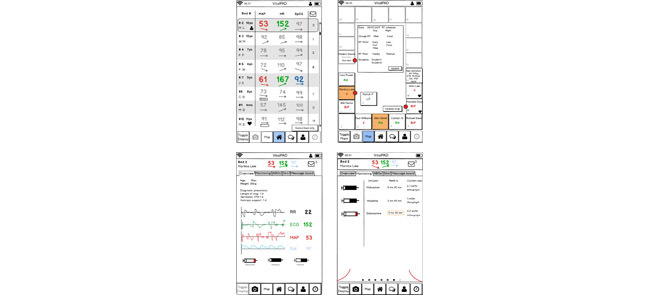

## I. Introduction

The pediatric Intensive Care Unit (ICU) can be a hectic, chaotic, and stressful work environment [1]. Clinicians are required to make difficult decisions about critically ill patients under the pressure of time. They need to continually observe, obtain, and evaluate a vast array of information and collaborate within a multidisciplinary team [2]–[5].

### A. Issues in the ICU

**FIGURE 1.**
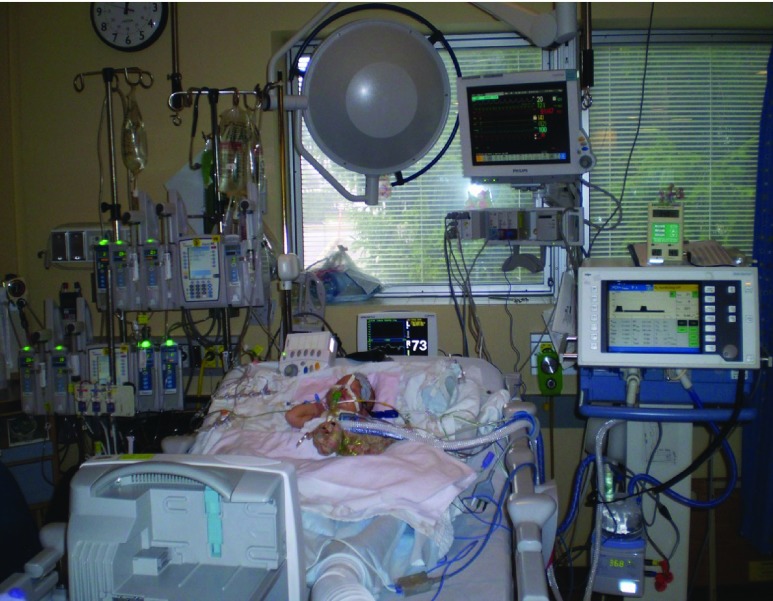
Typical bedside in the pediatric intensive care unit (ICU) (With permission of Heather van Mil, *http://lifeloveandthepursuitofplay.com*).

Data are obtained from multiple sources in different locations, e.g. through monitors and devices by the patient’s bedside (Fig. 1), by physically separated computers, or on paper in the patient’s chart [2], [6]. The amount of information can be overwhelming and difficult to process [7]. Accessing and integrating the required data for decision-making can be time-consuming and made difficult by multiple logins, the need to use different computers for certain tasks, or the information sources being occupied or otherwise unavailable. Furthermore, depending on local resources and structure of an ICU, a large amount of crucial information is still documented on paper by multiple team members, often redundantly. This can lead to missing or misplaced charts and a delay in flow of information.

Work efficiency and patient safety in the ICU depend on a well-functioning team. Doctors (MDs), registered nurses (RNs), and respiratory therapists (RTs) need to be up to date on patient status at all times, coordinate and follow plans for each patient, and closely communicate critical information. Even the most experienced team faces challenges by being physically separated and having to locate each other with pagers and phone calls [8], [9]. Since team members change with every shift, information can be lost in multiple handovers. In addition, quick patient turnover adds to the list of new issues that need to be resolved on a daily or hourly basis. The combined high cognitive workload and limited number of staff available at any given time leads to an overall decrease in situational awareness [10].

A review of 5017 patients from 8 cardiac and medical pediatric ICUs reported a new morbidity rate of 4.8% and mortality rate of 2.0% [11]. Root cause analysis of morbidity and mortality discussed in case conferences in pediatric ICUs identified unsafe processes or medical errors in up to 77% of cases [12], 60% of which were deemed to include some interface problem (including handovers, interdisciplinary discussions, and communication with families) as a contributing factor. Similarly, Cifra et al. found that preventable events included medication errors in 17.5%, communication/teamwork errors in 14.5%, workflow errors in 7.3% and diagnostic errors in 7.3% of cases [13].

### B. Current Technology Issues

Medical devices currently used in ICU settings are often poorly designed and their potential is not fully realized [14]. Although new technology can be a leading cause of preventable harm to patients in hospital [14], [15], well designed medical devices and technologies can improve patient safety and prevent adverse outcomes. The evolving introduction of modern technologies into healthcare provides an unprecedented opportunity to investigate how novel technologies can improve patient care [12].

Current issues with existing technology in ICUs have been extensively reviewed [16]–[18]. Challenges include the large number of devices and physically separated displays that overload users with information. Medical devices, including ventilators, infusion pumps, and other patient monitors, frequently do not interact with each other. Adding to cognitive workload, these devices often fail to convey information intuitively and lack context to support data interpretation; that is, values are shown without target ranges, which can vary widely based on treatment goals and individual patient status. For example, a monitor displaying blood pressure does not provide any information on what vasoactive substances are currently infusing. Alarms lack clinical context to support management decision-making and may not accurately represent patient state during interventions [19], [20]. The large number of false alarms in the ICU causes alarm fatigue and contributes significant stress to patients and care-providers [20], [21].

### C. Recent Advances

Recent progress has been made to address the technology and workflow issues in the ICU. For example, concepts have been developed for improving a neurological bedside monitor display, which highlights the importance of presenting data in dynamic and flexible formats to support clinicians from different disciplines in different clinical situations [22]; for assessing the use of an interactive visual aid to improve cardiac ICU team communication processes [23]; for evaluating an ICU-wide dashboard for tracking compliance with patient safety activities [24]; and for the design and preliminary testing of an electronic tool to improve the efficiency of ICU rounds [25].

Internet of Things (IoT) concepts and technologies are an area of significant research interest with potential applications in personal and institutional healthcare [26], [27], but still currently have limited implementation in nursing or ICU settings [28]. IoT technologies have the potential to facilitate patient monitoring, and improve quality of care and patient safety [28]. Indeed this potential was recently illustrated in an integrated Neonatology ICU (iNICU) with IoT-based collection of patient monitor, ventilator and blood gas machine data using Beaglebone devices [29].

In order to realize the benefits of IoT technologies such in a fast-paced and complex healthcare environment as the ICU, with diverse clinical roles and communication channels, it is important that development be driven by the clinicians’ understanding of their information needs and that the implemented technology fits existing workflows.

### D. The VitalPAD Application

*VitalPAD* is an application prototype, running on a mobile tablet computer, which combines information from multiple monitoring and therapeutic devices into one platform. By processing the data as an intelligent decision-support system, it aims to improve clinical decision-making, team communication, and situational awareness. Moreover, a reduction of cognitive workload may contribute to improvements in patient safety [6]. The primary focus of the *VitalPAD* application is to address communication and workflow issues, which may account for up to 22% of preventable errors in the pediatric ICU environment [13].

The application aims to provide ICU clinicians with an intuitive and integrated display of vital parameters for individual and multiple patients, smart alarms, and reminders, as well as a team communication tool. The application will display key parameters from every patient in the ward within one view, and will provide clinicians the option to view detailed information from individual patients in a separate window. It will also allow the user to select specific patients to display in the overview.

The objective is for the application to apply some intelligent processing to the integrated data. The processing will support clinical decision-making via smart alerts, cumulative risks scores and context-sensitive emojis/icons based on the assessment of vital signs and other monitoring data, drug infusion information, clinical checklists, and/or response to application messages. Monitoring data will be evaluated against user-configurable absolute thresholds, combinations of such thresholds, as well as relative changes over time. Furthermore, the aim will be to introduce predictive analytical models to identify vital sign changes known to precede patient deterioration [30]. Evaluating the feasibility of these proposed design features is beyond the scope of this preliminary application design phase.

### E. Aim of Study

The aim of this study is to identify requirements for the *VitalPAD* application and to design and evaluate application components through a participatory design process. Requirements are captured during clinician observation and interviews followed by iterative testing and improvement of potential application designs through testing the usability of application mock-ups. This project extends a previous work domain analysis (WDA) [31] of the ICU and rapid prototyping results [32]. This article will focus on the clinician-driven participatory design phase of the project.

## II. Methods and Procedures

Human research ethics board approval (H16–02361) was obtained. Certified MDs, RNs, and RTs were eligible to participate after providing written informed consent.

All clinicians were currently working in the pediatric ICU at BC Children’s Hospital, the only dedicated tertiary pediatric teaching hospital in the province, where general surgical, cardiothoracic surgical and medical pediatric patients are treated. Medical residents and other trainees were excluded. The typical clinician-to-patient ratio depends on the role and context. The typical nurse to patient ratio is one-to-one or one-to-two, but may be two-to-one for very sick patients. RTs typically take care of five to ten patients, depending on the staff situation, and MDs were responsible for every patient on the ward (maximum capacity of 22 patients).

The study consisted of two distinct parts to refine prototype requirements: a) clinician observation, and b) prototype improvement and usability testing. The use of a WDA and a participatory display design approach [33] ensured the active involvement of clinicians, engineering, and design experts in both phases.

### A. Clinician Observation

Observation was performed with detailed note taking to establish prototype requirements [34], [35]. A group of at least two researchers, with different scientific backgrounds (including one with clinical expertise), observed individual ICU clinicians and health care teams in their work environment to gather more information about ICU routines—how they work, communicate with each other, and make decisions regarding patient care.

Researchers were sensitive to clinical priorities during the observation sessions, but when appropriate, participants were asked to explain their actions and state their goals, with open-ended questions to clarify information needed for tasks. This approach aimed to find any perceived challenges and to collect suggestions for potential *VitalPAD* functions to improve decision-making and communication.

An ICU observation sheet was used to record data during these sessions. Information was recorded in the following categories: session information, handover, direct interaction with monitors, interventions, decision logistics, communication with team members, routine tasks, and interruptions. Specific areas were commented on within each category (Table 1). Notes were also recorded on any observed constraints, problems, or gaps in the flow of information.

**TABLE 1:** Observation Categories Reflecting Main ICU Activities

Topics	Sub-areas included
Session information	• Session date, time, and duration • Roles of clinician(s) present
Handover	• Handover structure (who was involved, where did it occur, how long did it take) • Information included in handover (clinical information about patient status and plan and non-clinical information)
Direct interaction with monitors	• Monitor manufacturing make and model • Frequency and reason for monitor viewing: e.g. to silence alarms, to view specific waveforms, to look at data trends
Patient intervention	• Any changes to ventilation, patient position, or drug and fluid administration • Why intervention(s) occurred (e.g. change in vital signs)
Clinical decision logistics	• Categories of information gathered and combined to make patient care decisions or interventions (e.g. visual patient status, vital signs, laboratory data, medication administration) • Use of data trends or what time frame of information was used (e.g. current values, or whether changes within seconds, minutes, hours, or days informed changes in patient care)
Team communication	• Who, when, and how team members communicated • When was face-to-face communication required • What information could possibly be conveyed in text format
Routine tasks	• What is considered a routine task • How long tasks took to complete
Interruptions	• Any interruptions to patient care and task completion • When, how, why, and by whom interruptions occurred

In particular, researchers observed handovers (end of shift, break coverage, and new admissions) and multidisciplinary rounds. This allowed the research team to determine what information clinicians required before making patient care decisions.

Additionally, researchers were also able to shadow clinicians while they trained other clinical/trainee staff (e.g. an RN training for certification to care for cardiac patients; a student RT). During these sessions, clinicians were already explaining their goals and actions to trainees.

At the end of the observation sessions, the researchers asked additional closed and open-ended questions, if time permitted. Example questions included: What do you think are the most important parameters on the monitor? Do you look at the trend view very often? How do you assess a patient’s status? What prompts you to intervene in the current treatment plan? What would make a mobile monitoring device useful for you?

Issues and possible solutions were summarized after careful interpretation of field notes and discussion between researchers. A template analysis approach was used to organize observations from field notes into the pre-determined themes identified as topics in Table 1; further feedback was sought from clinicians as required [33], [36].

### B. Prototype Improvement and Usability Testing

Design ideas were combined into an interactive portable document format (PDF) mockup using *balsamiq* (Balsamiq Studios, Sacramento, CA, USA). The previous rapid prototype, generated from the WDA in 2013, [32] was used as a foundation and design ideas derived from clinician observations were incorporated.

General feedback was obtained from the participating clinicians early on in this process, initially using paper printouts of the mockup, which were annotated and expanded upon. Clinicians then provided design-related suggestions and were shown different monitoring view options (e.g., current values, trends, artificial horizons) for feedback. The mockup design was improved accordingly.

Subsequently, five simple scenarios were created for usability testing and to refine requirements for prototype implementation. For each scenario, a question or set of questions or tasks were given to the participant to guide their review of the application mockups.

Scenario 1 (Ward Overview): Which patients in this scenario are likely to need your attention? Do the given designs provide you with enough information to get an overview of the ward, e.g. after you have been off-service for a week?
Scenario 2 (Vital Signs Monitoring): Do the given designs provide you with enough information about the patient’s overall status?
Scenario 3 (Reminder Setting): Use the reminder function to create a new reminder for your patient.
Scenario 4 (Brief Handover): Use the subscription function to cover another patient during a break.
Scenario 5 (Arterial Blood Gas Analysis Documentation): Take a picture of an arterial blood gas (ABG) analysis printout, annotate it, and share it with your team.

All scenarios were selected based on clinical relevance and to evaluate the core functions *of VitalPAD*. The initial explanation for each scenario, as well as the order the scenarios were completed, were standardized. Instructions and prompts during these sessions were kept to a minimum to investigate the intuitive nature of the design. However, while working through the task, some participants needed more guidance and the specific problem and reason for that was documented. The clinicians were also instructed to voice their thoughts during the process and all statements made were documented. Questions asked of participants were always related to the task completed and were intended to clarify what the participant was thinking. Two researchers conducted the usability testing, with one focused solely on documenting each interaction the clinician had with the mockup. Notes from the usability scenarios were analyzed to determine the frequency and perceived impact of the issues that were encountered, with the aim of identifying the most significant issues (most frequent and/or highest impact) to address during re-design of the mockup [37].

Mockups were displayed in an interactive PDF format on a tablet (Dell Venue 7, Round Rock, TX, USA) to make them resemble a real app. Clinicians asked questions and provided feedback throughout the scenario testing, and subsequently provided overall feedback.

## III. Results

### A. Clinician Observation

**FIGURE 2.**
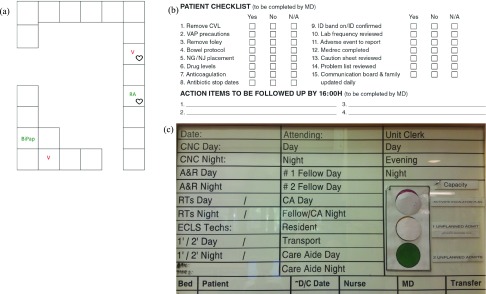
Examples of cognitive support resources currently used in the intensive care unit (ICU). The respiratory status map (a) is used by respiratory technicians and depicts patient rooms with each patient’s current level of respiratory support: Bi-level Positive Airway Pressure (BiPAP), Room air, Ventilated; heart icons denote cardiac surgical service patients. The patient checklist (b) is completed daily during rounds for every ICU patient: it includes mandatory and frequently used patient management and safety protocols. The central ICU staffing whiteboard (c) is located at the main nursing station and is completed daily by the ICU unit clerks and nurse in charge: it depicts who is responsible for each role during the day shift and night shift.

We observed a mixed sample of 10 clinicians (4 MDs, 4 RNs, and 2 RTs) for a total of 54 hours, including 8.5 hours of multidisciplinary rounds. During observation sessions, the existing cognitive aids used to optimize unit workflow were also documented. These were mostly paper-based and were updated daily. Examples included a nursing station white board; paper printouts of patient overviews and care plans for every team member; maps of patient respiratory status updated by hand; and additional documentation and checklists for each patient (Fig. 2). Observed and clinician reported problems with these systems included a) monitoring information overload and redundancy (e.g. multiple heart rate values obtained by different sensors attached to the same patient), b) redundant paper-based documentation (e.g. between nursing and RT flow sheets), and c) outdated communication methods with impacts on task completion (Table 2). Researchers did not explicitly quantify the number of patient monitor alarms, including false alarms.

**TABLE 2:** Observed Themes and Potential Opportunities for Software Implementation of $VitalPAD$ After Completion of Phase 1

Observed concerns	Possible solutions	Role(s)
Inefficient communication • Out-dated pager system • Staff not physically present in ICU or difficult to find • Redundant handover/updates	Text messaging system • Responses color-coded by clinical role for easy identification of sender • Team and/or personal messages instead of paging • Confirmation receipts for messages for closed loop communication	All staff
	Patient subscription • Select which patient(s) vitals to display on screen • Add additional patients to monitor when providing break coverage	RT / RN
Information overload • Multiple non-integrated monitors and therapy device displays (bedside monitor, ventilator, infusion system, etc.)	Integrated remote monitoring • Remote access to real-time data from all bedside devices • Integrated displays to reduce cognitive strain (e.g. combination of vital signs and infusion time to detect adverse effects)	All staff
Impaired task completion • Stressful environment with high workload (staff need to remember many tasks throughout the day) • Frequent interruptions make task completion more difficult	Smart alerts • Pre-set reminders and system alerts (e.g. pump XYZ empty in 30 min) to reduce cognitive load • Custom reminders (e.g. “call Dr. X to follow up results”) for tasks that do require completion at a later time	All staff
Redundant documentation • Paper-based charting of redundant information • Documentation of skin conditions or ABG via print-out, which get lost and are only available to one person at a time	Electronic support • Use device camera for photo documentation (e.g. ABG or skin condition), which will be saved electronically and can be sent to other team members • Annotate/message photos to team for feedback	RT / RN
Impaired situational awareness • Large patient load	Overview layouts • Map of ICU displaying simple trend views for quick assessment of each patient, which can help prioritizing patients	MD / Charge nurse
	Patient flow updates • Alert staff about new admissions and transfers • Display which beds are available	All staff
	Integrated trend views • Early recognition of possible adverse events	

RT=respiratory therapist, RN=registered nurse, MD=medical doctor, ABG=arterial blood gas.

Researchers attended three morning rounds and two afternoon rounds (shorter and less detailed than morning rounds). For all RNs and RTs observed, researchers witnessed their end of shift handover as well as break handovers.

#### 1) ICU Rounds

During the observation period, all health professionals involved in that patient’s care attended interdisciplinary ICU morning rounds. Attendance ranged typically from five to ten participants, but was over twenty for cardiac surgical patients. The team often also included non-clinical staff, such as researchers or ethicists. The team gathered at each patient’s bedside or around a central computer if reviewing x-rays or other imaging studies. At the bedside, the patient’s nurse, RT, and ICU fellow (a senior trainee MD) verbally presented current status and overnight events to the team. The fellow summarized ongoing problems and the team discussed possible action items. Individual team members verbally contributed ideas to the plan and used personal cellular phones or bedside computers to access medical resources. Before the team proceeded to the next patient, a resident (a junior trainee MD) or ICU staff physician (attending MD) recorded the physician orders on paper, and a patient checklist (Fig 2b) was completed.

#### 2) Concerns Identified During ICU Rounds

Verbal discussion during morning rounds was challenging to hear and understand. The individual speaking was often up to five meters away from team members due to the team size and open-ICU format.

Ambient ICU noise and concomitant discussion amongst individual team members interfered with understanding. Bedside alarms were frequently silenced. For cardiac surgery patients, team members often moved closer to the patient during rounds in order to properly hear the discussion. The ICU staff physician reviewed nursing flow sheets and approached bedside monitors to look at current vitals and vital sign trends. None of this information was easily viewable to the rest of the team unless they physically moved to look at it.

#### 3) Handovers

End of shift handover for bedside RNs was one-to-one at the bedside and covered only their assigned patients. The charge nurse and MD handovers were also usually one-to-one, but covered all ICU patients. Charge nurse-MD handover usually occurred near the main nursing station using the central ICU whiteboard with patient and staff assignments (Fig. 2c), or using the patient information summary table prepared by MD residents each morning. The summary table provided a brief bullet-point overview of each patient with headings for patient information; past medical history; active issues; systems overview (sedation, respiratory status, medications, feeds, lines, microbial cultures); important dates (admission, intubation, extubation, procedures), and the treatment plan. All RTs (night and day shift) met at shift turnover and discussed each patient with emphasis on those requiring respiratory support. They also had a binder containing admission information and daily updates for each patient.

#### 4) Issues Identified During Handovers

RNs conveyed information at end of shift handover systematically using the nursing patient flow sheet and commented on diagnosis, respiratory or oxygen support, feeding, recent laboratory results, current issues, and plan for the day. Handover was briefer between nurses whom were already familiar with the patient and focused on more recent information; conversely, handover to a nurse unfamiliar with the patient took longer. Nurses conveyed as much information as possible during the limited time for handover, afterwards clarifying and researching further details in the patient’s chart. One RN was seen to verify recorded drug dosages and vital sign values after shift handover to ensure handover information had been correct.

The bedside nurses assigned to adjacent patients often covered each other’s breaks. Due to the physical distance between patients, or patients being located in single-patient rooms, it was difficult for nurses covering two patients to consistently view patient monitors.

Handover between RTs was frequently delayed due to difficulty locating each other in the building.

#### 5) Clinician Comments

Observed clinicians were asked how they thought a mobile application could help in the ICU and what ideas they hoped the design would include. Five clinicians (5/10) wanted an easier method of contacting team members for scheduling breaks, for asking minor questions or making requests, and for sending brief patient updates. Three MDs (3/4) stated they use central vital sign monitors regularly; for example, to review vital signs leading up to cardiac arrest or other adverse events and wanted a more user-friendly way of monitoring patient vital signs and trends when away from the bedside.

### B. Usability Testing and Prototype Improvement

Initial prototype design ideas were generated from workflow observations, identified issues, and clinician feedback (Table 2). These ideas included: integrated displays customizable to the clinician’s needs, which would allow the user to select patients shown in the overview display, with the aim of improving situational awareness and facilitating the identification of sicker patients; pre-set and custom reminders to help reduce the heavy cognitive load ICU clinicians face with daily task burdens; a photo-documentation option to allow immediate communication of print-out results, compared to the existing system, in which an individual clinician is notified of the result and others must access the paper chart for the information; and a communication system color-coded by clinician role to reduce the need for outdated pager systems and improve team communication through streamlining correspondence onto one central platform. Regular meetings among study researchers were held to discuss ideas and their feasibility. Some features requested by clinicians, such as quick viewing of a patient’s active orders or medications and laboratory results, were documented, but ultimately unlikely to be feasible. Feasibility of design ideas was limited due to several factors: 1) institutional constraints and privacy issues (e.g. the inability to access laboratory data or the electronic medical record), 2) legal issues (*VitalPAD* cannot replace the medical record), 3) technical limitations, and 4) user characteristics (members of the health care team’s experience and preferences in interacting with a monitoring device).

Design ideas were formulated into a prototype based on the original 2013 WDA prototype. Feedback on the prototype from a total of 15 clinicians (5 MDs, 6 RNs, 4 RTs) was acquired in two ways: through general feedback conversations and through feedback from participants completing the usability scenarios.

#### 1) General Feedback

Seven clinicians (3 MDs, 2 RNs, 2 RTs) were interviewed for general feedback on the prototype created after the observation phase. Interviews ranged from 15 minutes to an hour in length, based on the clinician’s availability. Feedback reported here reflects the most common comments and is organized in order of comment frequency.

**FIGURE 3.**
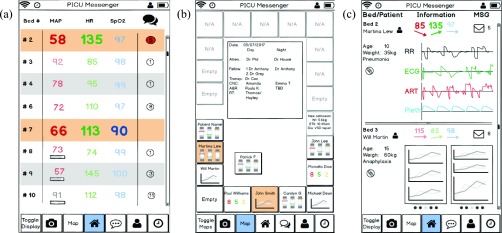
Examples of early prototype designs that were rejected by clinicians. For example: the minimal patient list overview (a) did not provide relevant information such as age or patient initials; the ward and staffing overview map (b) received positive feedback, but clinicians did not like the alternative display options of patient status, including artificial horizons; and the patient monitoring overview layout (c) contained helpful information, but a different format was preferred, with information tabs for one patient at a time (see Fig. 6).

**FIGURE 7.**
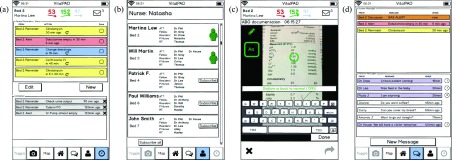
Examples of screens used for reminder, handover and arterial blood gas (ABG) (scenarios 3, 4 and 5). Using the reminder screen (a), clinicians were asked to create a new reminder (e.g. check urine output) for their patient. Using the subscription list screen (b), clinicians were asked to subscribe to a new patient in order to cover that patient for a colleague’s break. Using the ABG camera and annotation screens (c), clinicians were asked to take a picture of an ABG analysis paper printout, annotate it, and send it to the team using the communication function (d). The top section of the communication screen (d) shows patient alerts such as new ABG and reminders. The bottom section (d) shows personal messages between clinicians.

All clinicians (7/7) agreed that combining information from different bedside devices and other sources would help support clinical decision-making. The option to view patient data from monitoring and therapeutic devices remotely was identified as the application’s core beneficial feature. The overview list, including vital parameters and simple trend indicators (Fig. 3a) as well as the map view of the ward (Fig. 3b), were considered very helpful, especially when caring for several patients at once. Five (5/7) clinicians said they view patient trends to understand previous patient status. Additional features allowing vital sign and drug infusion data integration (Fig. 3c), creation of reminders for task completion, direct messaging between clinicians and team members subscribed to a patient (Fig. 7d), and options to photo-document ABGs and skin lesions also received positive feedback.

Critical feedback about the prototype was also received. All clinicians (7/7) mentioned the importance of role-specific designs. Overview screens with artificial horizons and a cumulative risk score (Fig. 3b) were shown to 4 clinicians in the first round of feedback and were rejected by all of them: they were hesitant to rely on a cumulative risk score that is unfamiliar to users and were concerned that understanding artificial horizons would require additional training. Thus, these two features were removed from the prototype before completing the feedback round. Clinicians instead preferred re-use of designs already present in the ICU; five (5/7) indicated that they favor real-time numbers and graphs so they can apply their own experience and knowledge for interpretation; and two (2/7) favored common color codes for vital parameters. Layouts that were too minimalistic (Fig. 3a) or too crowded (Fig. 3c) were also rejected. They preferred to pick and choose which patients are displayed in overview layouts: a physician in charge of the whole unit has different monitoring needs than a nurse looking after one patient. One clinician asked how *VitalPAD* would alarm (would there be pop-ups within the application?) and whether it would replace bedside monitor alarms.

Clinician feedback was summarized (Table 3) and the prototype revised. Design elements that received mixed feedback were given particular consideration and efforts were made to accommodate all viewpoints. For some, we were able to modify the mockup to incorporate all feedback: for example, in the drug graph, some clinicians preferred being able to see multiple drugs, others found it too confusing and just wanted to see one drug; the identified solution was to allow users to select the number of drugs per graph. For others, we followed the majority opinion for the primary display, but had the other information accessible elsewhere in the app: for example, to address whether to display age vs weight; we show age on the ward overview screen, but include weight in the patient overview.

**TABLE 3:** Detailed Clinician Feedback of General Sessions, First Part of Phase 2

Design	Feedback
List overviews (Fig. 5)	• Three most important vital parameters: mean arterial blood pressure (MAP), heart rate (HR), oxygen saturation (Sat) • Trend arrow should cover last 30–60 minutes • Patient’s age or weight important • Colors should match the currently used bedside monitors • List with detailed patient information good to keep everyone on the team up to date • Screen appears cluttered • Indicator for non-invasive blood pressure not clear
Map overviews (Fig. 5)	• Supports geographic orientation of the ward • Responsible staff for each patient easy to find • Map for respiratory status could help with patient care planning • Map with smiley indicators too vague, not clear what it stands for • Abbreviations need to be changed to meet local standards
Monitoring Information (Fig. 6)	• All vital parameters displayed on the monitor need to be included • Having waveforms visible is important for ECG and invasive BP • A trend view with a slider for past events is very useful for MDs • Detailed ventilator information very useful for RTs and MDs • Infusion pump information very useful for RN • Drug trends and combined flow charts confusing to look at
Photo Documentation (Fig. 7c)	• Arterial blood gas analysis (ABG) printouts get lost or are difficult to pass on to the requesting clinician, option of recording electronically as a picture is very helpful • Annotation tool useful if no personal communication possible • Indicator needed that the receiver has seen it • Keep ABG pictures for 1–2 days only
Communication tool (Fig. 7d)	• Good for non-urgent communication • Could add to number of alerts received by care provider

RT = respiratory therapist, RN = nurse, MD = doctor, ABG = arterial blood gas.

#### 2) Scenario-Specific Feedback

**FIGURE 4.**
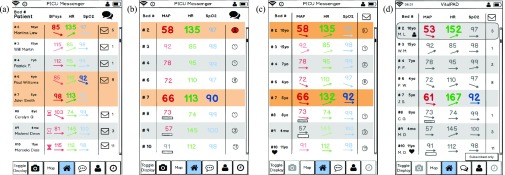
Evolution of patient overview list based on clinician feedback, illustrating the change from initial prototype (a) through interim designs (b) and (c) to final prototype (d). The simplified prototype (b) did not include enough information for clinicians, who requested re-addition of trend indicators, and patient initials, and age. Non-invasive blood pressure monitoring was differentiated from invasive measurements using small rectangles indicating the age of intermittent measurements; this design received favorable feedback. Design (c) re-introduced trend arrows and patient age, changed the icon for messages, and added heart icons to indicate cardiac surgical patients. The final design (d) re-introduced patient initials, further simplified the messaging icons, and highlighted in color any critical values reflecting a change in patient status. Values not reflecting a significant change were grayed out. Finally, a small person icon was added to indicate patient(s) to which the user is subscribed.

The usability scenarios with the interactive PDF mockups were completed by eight participants (2 MDs, 4 RNs, 2 RTs) over two rounds. Clinicians took between 15 and 30 minutes to complete all scenarios. The mockup prototypes were improved after each round in response to the feedback given; for example, see Fig. 4 for versions of the patient overview list, which improved with clinician feedback. After completing the usability scenarios, clinicians were asked for overall feedback. The most frequent comments from both feedback rounds are summarized below.

##### a: Scenario 1 – Ward Overview

**FIGURE 5.**
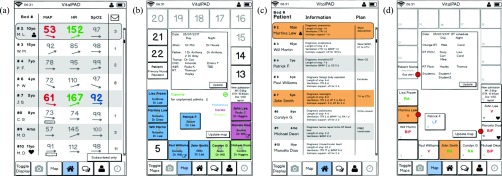
Examples of screens used for the ward overview (scenario 1). Using these four screens, clinicians were asked to identify which patients are likely to require attention and whether the designs provided enough information to understand overall ward status. The overview list (a) shows patient initials, age, current vital signs and messages. The map overview (b) depicts patient location, nursing and physician assignment, and responsible specialty; ICU capacity and overall staffing. The patient information list (c) includes information found on the existing physician summary sheet: diagnosis, length of stay, antibiotic treatment duration, length of intubation, level of respiratory support and a brief management plan. The respiratory status map (d) was modeled on the existing status map (see Fig. 2a) and shows respiratory therapist staffing, patient respiratory status and red circular alerts for new arterial blood gas measurements.

For the overview scenario, clinicians were asked to identify which patients were likely to need immediate attention and asked whether the design provided enough information to form an overall impression of the ward. All clinicians (8/8) were quickly able to identify the patients with abnormal vital parameters in the list overview (Fig. 5a). In combination with the map overview (Fig. 5b) and the more detailed patient list (Fig. 5c), all clinicians agreed that they would have a comprehensive initial impression of the unit. While the displays provided the required information, 2/8 clinicians found the font size used in the map views challenging to read.

Opinions varied on which parameters should be displayed on the patient overview list. MDs emphasized the importance of displaying the systolic blood pressure (SysBP) instead of mean arterial pressure (MAP) for cardiac surgical patients. However, for the majority of ICU patients, MAP was most relevant. One RN suggested displaying weight instead of age. The prototype was revised based on these suggestions (Fig. 4).

For the patient status map, opinions varied on what the emojis (e.g. sad or happy faces) should signify. Two clinicians felt the emojis should indicate whether a patient is “sick” versus “not sick.” Two other clinicians felt the emojis should indicate level of care according to existing PICU level of care classification protocols. One clinician felt the emojis would be most helpful if they indicated whether the patient is ready for transfer.

The respiratory status map (Fig. 5d) was considered useful, but 6/8 clinicians had difficulty finding the legend for notification indicators (e.g. “gas alert” signifying a new ABG result). To address this difficulty, the legend was moved to the center of the screen, and the shape and background were changed. The new legend features a smooth rounded corner border and is light grey, providing greater contrast from the square white patient rooms.

##### b: Scenario 2 – Vital Signs Monitoring

**FIGURE 6.**
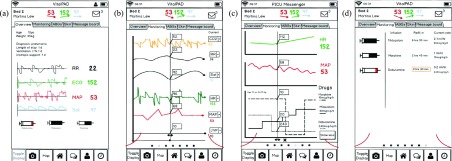
Examples of screens used for vital signs monitoring (scenario 2). Using these four screens, clinicians were asked if the given designs provide them with enough information to discern overall patient status. All screens feature a header bar with the patient bed number, name, current mean arterial pressure (MAP), heart rate (HR) and oxygen saturation (Sat), and messages. The patient overview tab (a) shows the patient age, weight, diagnosis, length of stay, and how long they have required inotropic or respiratory support; current waveforms for respiratory rate (RR), electrocardiogram (ECG), MAP and Sat; and drug infusions. The drug infusion icons depict volume of syringe remaining. The monitoring trends tab (b) shows trends of various parameters: end-tidal carbon dioxide (CO2), RR, Sat, HR, MAP and jugular venous pressure (JVP). The integrated vitals and drug infusion display (c) shows HR and MAP versus current drug infusions. The drug infusion status screen (d) shows which drugs are currently infusing, the current infusion rate and when syringes need to be refilled.

For the monitoring scenario, clinicians were asked if the designs provided enough information to discern a patient’s current status. 8/8 clinicians used the tab style design (for the various types of patient information) with ease. Clinicians considered age, weight, and diagnosis in the “overview tab” (Fig. 6a) before moving on to the “monitoring tab” (Fig. 6b). All clinicians reported the designs to be self-explanatory and easy to navigate. 3/8 clinicians were critical of the integrated drug graph (Fig. 6c), noting that it did not indicate the previous infusion rate, or an option to pick a certain drug or group of drugs to display in the graph. One RN mentioned that the device would not be as useful for bedside nurses because they are always within eyesight of their patient and do not need real time updates on other patients. However, this clinician stated the communication functions and subscription functions were helpful.

##### c: Scenarios 3 and 4 – Reminder and Handover

For the reminder and handover scenarios, clinicians were asked to create a new reminder for a patient and subscribe to a different patient that they were not currently following (Fig. 7a & 7b). All clinicians (8/8) completed both tasks without major issues. Two clinicians (2/8) were unsure which button led to the subscription function. One clinician (1/8) selected the clock icon on the app header toolbar, instead of using the clock navigation button to set a reminder. In response, we changed the analogue clock on the display header to show the time in digital format. The icon for subscription (a person) was not changed because the two clinicians agreed, after a brief explanation, that the icon was appropriate for the corresponding function.

##### d: Scenario 5 – Arterial Blood Gas Analysis Documentation

Currently, ABG results are printed on a small slip of paper and attached to the patient chart. For this scenario, clinicians were asked to take a photo of this printout, annotate it, and send it to the team (Fig. 7c). 4/8 clinicians completed this scenario without significant guidance. Some clinicians had difficulty finding the correct button for annotation and instead clicked on the guidance text (“Annotate ABG”), asking how to use the pencil and text tool included in the mockup. Annotation and text addition functions were limited by the PDF format of the mockup; for example, participants attempted to use a finger to circle a value but this action was not supported by the mockup and they were re-directed to click the “circle” button.

After the first round of usability testing, a display showing all previous ABG recordings was added. The display originally had the oldest ABG file displayed at the top, but was updated after the second round as one clinician strongly felt the newest record should appear first.

##### e: Overall Feedback

After completing the usability scenarios, clinicians provided overall feedback. Clinicians were asked an open-ended question regarding their general thoughts of the application and ease of use. Six clinicians described the prototype as easy to use and helpful. However, meanings of buttons were not always intuitive to first time users. Once explained, these functions became easy to learn. An online tutorial prior to use or a teaching session for participating staff could help familiarize the users with the layout.

## IV. Discussion

The ultimate aim of this study was to design a mobile monitoring and communication application that will assist clinical decision-making in the ICU. The design approach required the active involvement of clinicians, with the aim of creating an application that fits their workflow and is tailored to their needs. The initial phases of observation and usability testing provided useful insight into the daily functioning of the ICU and a wide array of ideas for implementation of the prototype design. The research team itself included people with clinical expertise and this likely improved the efficiency of the requirements gathering process and, in particular, facilitated effective communication with ICU clinicians.

Participatory design approaches have significant potential to facilitate the design and implementation of healthcare technology [38]. They have been applied effectively in a range of healthcare settings for the development of patient tools, clinician tools, and health information systems. For example, they have been used in health departments [39], hospital clinics [40], mobile [41], and rural [42] environments, as well as in the ICU [43]. While these examples demonstrate the success of participatory design, these applications largely have not been safety critical monitoring tools, using IoT-enable technologies for data capture. Notably, the recently developed iNICU uses IoT methods to record data from patient monitors, ventilators and blood gas machines [29]. This work shows that participatory design is an important approach in the design of safety critical ICU tools, where user engagement can lead to a more effective and ultimately better accepted design.

The observations confirm that the ICU is a complex and stressful environment. Information overload and redundant documentation decrease situational awareness, which may impair patient safety [44]. Modern technologies are available, but are often not effectively utilized or may even contribute to the clinician’s workload; for example, at morning rounds, clinicians interacted with bedside displays independent of the rest of the team. These displays were challenging for other clinicians to view concurrently because of small display size and viewer distance from the display. During interviews and feedback sessions with ICU clinicians, it became clear that a monitoring device that combines multiple data sources and supports clinical decision-making and team communication would be welcomed and could offer potential solutions to decrease mental workload. This is in keeping with previous work on ICU displays and device integration [45]. Functions for team communication also have the potential to reduce reliance on outdated systems such as numeric pagers, decrease time spent locating team members, and improve communication efficiency overall.

Researchers did not quantify the number of alarms but observed clinicians quickly silencing or disregarding them. This observation is consistent with previous reports of device alarms in the ICU [6], [46]. During feedback sessions, one clinician asked how *VitalPAD* would alarm and whether or not it could replace existing bedside systems. *VitalPAD* is not intended to replace bedside alarms, but rather to complement them. As a patient safety measure, these would remain active, but thresholds could be set such that they only alarm in urgent situations.

### A. Importance of Clinician-User Engagement

Observation in situ is crucial to understanding workload, cognitive and communication demands as well as system complexity. Individual clinician observation provides insight into each specific role, whereas observation of team rounds and handover inform how data are integrated and how clinicians interact. Although much can be learned from observation alone, interviewing and engaging clinicians in the design process is also required. Involvement in the iterative prototyping process was crucial in creating a functional design and will likely help with application implementation in the long-term. Although members of the design team have clinical experience, ICU clinicians are experts in the day to day functioning of the ICU and must be consulted.

IoT-enabled technologies offer huge potential benefit in hospital settings; these environments are rich in data, but in many cases it is currently not well-integrated. There is likely to be a significant growth in the use of these technologies to reap the benefits of these data to improve healthcare decision-making [26], [27], and it is therefore important that robust approaches are adopted for optimal development and implementation. It is estimated that 15% of technology projects are abandoned and more than 50% of the developers’ time is spent reworking the design after implementation [47], [48]. One goal of the user-centered approach is to ensure fewer failures by capturing and mitigating potential design flaws early in the development process [33]. Furthermore, in other settings, designer-or technology-driven innovations can be introduced with some re-design of working practices and workflows, but this is much less possible in clinical environments. Safety is the primary concern, overriding other potential benefits such as ease-of-use and cost savings. To minimize potential errors, new technology should fit established working patterns and often benefits from adopting features of existing systems. The risks of failure are significant and it is crucial that experienced clinicians should be involved at an early stage in the validation of the new system [49].

Designs such as the artificial horizon and cumulative risk score were introduced by engineering designers but ultimately rejected by clinicians, because of concerns about unfamiliarity and potential learning overhead, while a number of suggestions were made for emojis to denote patient status. This suggests that intelligent application features must be transparent to the expert user and should be considered for introduction as part of a staged approach.

Feedback obtained from the participants has been generally positive and constructive, acknowledging that the suggested prototype contains innovative ideas and is easy to use, but also takes time to adjust to. Clinicians were very engaged during the project, identifying potential problems with the prototype, recognizing challenges in their daily work, and providing creative solutions. One RN participant did not think the device would be as useful for bedside nurses because they are within sight of their patients at all times.

The iterative design process using *balsalmiq* mockups enabled rapid feedback. Because of this technique, we were able to better engage clinicians and efficiently modify our prototype. We learned that showing clinicians variations of each design page worked well in regards to user-engagement and generated suggestions for improvement.

Ethnographic studies of clinical end-users interacting with medical devices and clinical information systems [10], [35], [50]–[55], are becoming a frequently employed method to guide the participatory design of future patient monitoring and decision support tools [10], [31], [32], [35], [56], [57], demonstrating the value of this mixed development approach.

### B. Setting Specific Considerations

Our ICU observations and experiences prototyping displays for the *VitalPAD* have wide applications both within and outside the ICU. Effective team communication is crucial for hospital-based patient care. User-friendly messaging systems, as included in *VitalPAD,* could improve communication amongst medical and surgical ward teams in addition to adult and neonatal ICU teams.

Mobile monitoring of vital signs and therapy parameters (including infusion data) can help improve patient safety in any ward based on single-patient rooms. Currently, clinicians must enter patient rooms to see ventilator and infusion pump settings and alarms. Newer ICUs are increasingly being set up with each single patient room fully enclosed by walls, which potentially introduces risk [58]. Easily accessible patient information on a mobile device can help streamline workflow for all clinicians and prevent unnecessary trips to the bedside [7], [10], a phenomenon occurring in all inpatient settings.

Photographic recording and team messaging of ABG results could be useful for post-operative patients or any scenario where ABG results guide patient management.

The design needs to acknowledge setting-specific considerations, including differences in patient population, staffing ratios, and the different range of comorbidities encountered in adult and pediatric ICUs [32].

### C. Limitations

Although *VitalPAD* may improve communication and streamline clinical workflow, clinicians will still have to access institution computers and paper charts to view laboratory results, active orders, and medications. Incorporating these functions into *VitalPAD* may be desirable, but institutional constraints, privacy requirements, and legal concerns limit feasibility. *VitalPAD* is a tool for ICU clinicians to quickly access patient information, but its data will ultimately not be included in official medical records. It is likely that *VitalPAD* can expand to realize its full potential, but integration with electronic medical record systems may be a challenging process and thus remains a long-term goal. This system integration will likely require widespread acceptance of *VitalPAD* in the pediatric ICU, in addition to ongoing support by health records management, hospital administration, biomedical engineering, and clinical information technology teams.

For *VitalPAD* to be effective as a rapid communication system, all clinicians must have access to a tablet device and respond to system alerts. *VitalPAD* alerts must be appropriate and well designed to not add to the already significant false alarm burden. Carrying an additional device, which is primarily dependent on touchscreen interaction, may not be practical for all clinicians in all situations and may even have some implications for infection control; options for auditory and vibro-tactile notifications should be considered.

The overall workflow of the *VitalPAD* application has not been fully specified and remains a work-in-progress. This will not be straightforward, as even a single process, such as patient handover, may vary by role, by clinician, by clinical situation. The application will need to support clinical practice without placing over-bearing constraints on how the work is done [59]. It must also be recognized that adding a new device into a clinician’s workflow introduces a potential new source of error. Hence, our adoption of a participatory design approach to elicit maximum feedback from the end-users. To mitigate errors of this kind, appropriate training must precede the application’s use in a clinical setting.

Finally, we must acknowledge the limited scope of this study. We have refined the application requirements and established some preliminary designs, which are acceptable to the target users. We have not yet investigated the feasibility of these designs with respect to technical implementation or clinical practice nor evaluated the potential impact of the *VitalPAD* on improved communication, workflow, responsiveness to patients’ needs, or clinical outcomes. These will need to be addressed in future work.

### D. Future Work

Upcoming project phases include further clinician feedback and prototype revision.

Triaging experiments using realistic ICU patient scenarios will be conducted to further test usability in a simulated clinical setting. These experiments will investigate whether clinicians can identify and prioritize patients requiring immediate attention using the information displayed on the *VitalPAD* compared with conventional presentation on monitor printouts. We plan to conduct this experiment with MDs, RNs, and RTs; each participant will triage a number of patient scenarios, using either the *VitalPAD* or conventional displays. Both time to triage and correlation with expert consensus will be evaluated. We will also endeavor to assess mental workload, acceptability and user perception.

Discussion is ongoing between researchers, engineers, and clinicians about additional device integration, such as extra-corporeal membrane oxygenation (ECMO), and how *VitalPAD* could further interact with existing electronic health systems.

*VitalPAD* alert and notification design is also ongoing. Alarms are unlikely to be threshold based, due to their design limitations [18], [60], [61]. Future work will likely include design and implementation of smart-alarm systems, allowing earlier detection of at-risk patients and prediction of undesired outcomes. Future work may include investigating whether *VitalPAD* could be implemented on personal smartphone devices.

Finally, we will need to evaluate the prototype application in a real clinical setting. We propose implementing the *VitalPAD* in two beds within the BC Children’s Hospital pediatric ICU. Interfaces to ICU medical devices will be implemented, including the server infrastructure for vital signs aggregation, creation of reminders, and exchange of information. Clinicians will be trained and given the *VitalPAD* application on a handheld device. We aim to assess whether the *VitalPAD* will reduce bedside device alarms, reduce the number of pager calls used to seek advice or initiate activities (and replace them with messages through *VitalPAD*), increase alarm effectiveness, decrease alert response times (e.g. time to change ventilator settings), and improve timeliness of scheduled drug administration.

## V. Conclusion

Based on feedback and our observations, clinicians require a user-friendly application that portrays essential clinical information and generates a mental image of patient status at a glance. They require a system that facilitates quick communication, enables seamless patient handover, and permits reminders for various clinical tasks, such as infusion re-fill times.

If successful, this integrated system could enhance performance of the clinician in real-time, optimize therapeutic interventions, reduce adverse outcomes, and ultimately save lives. This project is ongoing and further research, critical feedback, and prototype improvement are necessary to ensure usability and feasibility of *VitalPAD*.
